# The impact of bisphenol A on the placenta^†^

**DOI:** 10.1093/biolre/ioac001

**Published:** 2022-01-08

**Authors:** Enoch Appiah Adu-Gyamfi, Cheryl S Rosenfeld, Geetu Tuteja

**Affiliations:** Department of Genetics, Development and Cell Biology, Iowa State University, Ames, IA, USA; Department of Biomedical Sciences, University of Missouri, Columbia, MO, USA; Data Science and Informatics Institute, University of Missouri, Columbia, MO, USA; Thompson Center for Autism and Neurobehavioral Disorders, University of Missouri, Columbia, MO, USA; Department of Genetics, Development and Cell Biology, Iowa State University, Ames, IA, USA

**Keywords:** BPA, trophoblast, placenta, pregnancy complications

## Abstract

Bisphenol A (BPA), an endocrine-disrupting chemical, is used to produce a wide variety of plastic and common house-hold items. Therefore, there is potential continual exposure to this compound. BPA exposure has been linked to certain placenta-associated obstetric complications such as preeclampsia, fetal growth restriction, miscarriage, and preterm birth. However, how BPA exposure results in these disorders remains uncertain. Hence, we have herein summarized the reported impacts of BPA on the morphology and metabolic state of the placenta and have proposed mechanisms by which BPA affects placentation, potentially leading to obstetric complications. Current findings suggest that BPA induces pathological changes in the placenta and disrupts its metabolic activities. Based on exposure concentrations, BPA can elicit apoptotic or anti-apoptotic signals in the trophoblasts, and can exaggerate trophoblast fusion while inhibiting trophoblast migration and invasion to affect pregnancy. Accordingly, the usage of BPA products by pregnant women should be minimized and less harmful alternative chemicals should be explored and employed where possible.

## Teaser

This review provides insight into the mechanisms by which bisphenol A, an endocrine-disrupting chemical that is used in plastic found in common household items, affects placental development, potentially leading to pregnancy complications.

## Graphical abstract

**Graphical Abstract:**
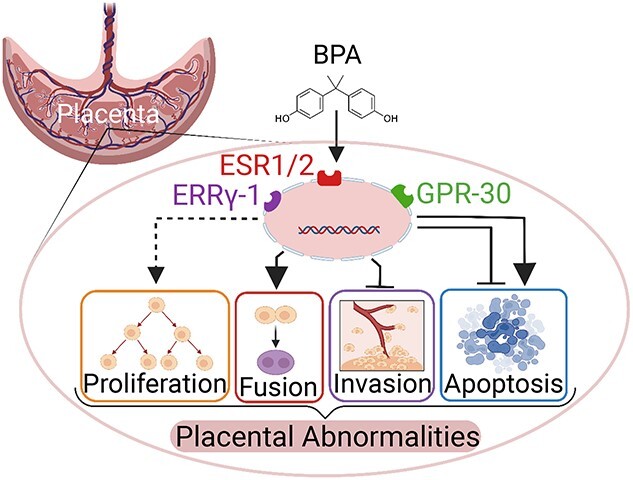

## Introduction

The success of pregnancy is largely dependent on placentation, a process that begins immediately after implantation of the blastocyst into the uterus and continues until a mature placenta is formed to mediate the physiological communication between the mother and the developing fetus [1]. In mice, the mature placenta is made up of three main layers: the labyrinth, the junctional zone that includes the spongiotrophoblast and parietal trophoblast giant cells, and the decidua [2]. In humans, cytotrophoblasts (CTBs), syncytiotrophoblasts (STBs), and extravillous trophoblasts (EVTs) are the major trophoblast subtypes that constitute the mature placenta, with the CTBs serving as the progenitor cells of the STB and EVTs. Proliferation and fusion of the CTB cells form the STB layer, while the proliferation of the CTBs and their subsequent acquisition of mesenchymal properties lead to the formation of the distal column trophoblast (DCTs), which are the only EVT lineage found in the first trimester placental villi [1, 3]. Once formed, the DCTs migrate into the decidualized uterus, becoming interstitial EVTs which subsequently remodel the uterine glands (as endoglandular EVTs) and the spiral arteries (as endovascular EVTs). Excessive trophoblast invasion is prevented via the fusion of the interstitial EVTs to form noninvasive cells called giant cells [1, 4]. For further details on placenta architecture and a diagram of the major cell types in mouse and human placenta, the reader is referred to other review articles [5, 6].

As placentation progresses, superfluous, infected, damaged, and/or dysfunctional cells are eliminated through apoptosis. Therefore, trophoblast proliferation, fusion, invasion, and apoptosis all contribute to placentation [1]. Each of these processes is regulated by several transcription factors and signaling pathways, and their impairments often lead to pregnancy complications, such as preeclampsia, miscarriage, and fetal growth restriction [7–14]. Hence, the presence of xenobiotics (foreign substances), in particular those that can exert endocrine-disrupting effects, in the placenta can interfere with trophoblast cell fate decisions to impair placentation, and lead to pregnancy complications.

One xenobiotic that has gained attention in female reproductive health is bisphenol A (BPA). This chemical is a common additive of plastics, epoxy resins, and other polymeric materials. It is used in the production of food and beverage containers, medical equipment, adhesives, coatings, water pipes, high-performance composites, and automotive and aircraft parts [15]. Bisphenol A is also prevalent in the environment. Diet is considered the primary route of exposure in humans and other species, but it may also be absorbed through the skin [16, 17].

It has been reported that BPA is an endocrine-disrupting chemical (EDC) which binds to nuclear estrogen receptors (ERs) and acts as an agonist as well as a selective ER modulator. Thus, BPA is able to mimic the actions of estrogen [18]. In adults, BPA is largely metabolized through glucuronidation; nevertheless, an unconjugated percentage can reach target tissues of the body [19]. In some tissues, the inverse process of deconjugation can take place [20]. Since the production of BPA continues to increase astronomically, with current rates at about 20 billion pounds per year [21], the adverse health effects associated with its exposure are bound to increase exponentially.

Global studies have revealed the blood levels of BPA in pregnant women from different geographical settings. For instance, in German pregnant women, the levels of circulating BPA were found to range from 0.3 to 18.9 ng/ml; and in the term placenta, the levels were reported to range from 1.0 to 104.9 ng/g [22]. In southeastern Michigan mothers, maternal blood levels of BPA were observed to range from 0.5 to 22.3 ng/ml [23], while in Korean pregnant women, blood levels of BPA ranged from nondetectable levels to 66.48 μg/l, and from nondetectable levels to 8.86 μg/l in cord blood [24]. Findings from several experimental studies indicate that even BPA concentrations within the pico and nanomolar ranges (extremely low concentrations) are able to exert adverse effects on the body [18, 25]. It has been found that elevated maternal blood levels of BPA associate with the occurrence of pregnancy complications [26, 27]. However, the molecular mechanism by which BPA leads to these pregnancy complications has not been established.

Since pregnancy complications often result from abnormal placentation [7–13], several studies have investigated the effects of BPA on placental formation. While some of these studies involved the use of trophoblast-derived cell lines such as BeWo, JEG3, and HTR-8/SVneo cells, others employed primary trophoblasts and animal models. Although these models do not fully mirror in vivo human placental conditions, their use is deemed acceptable since it is unethical to experimentally expose pregnant women to BPA. It is the findings from these studies that we have aggregated in this review. The overarching goal of this review is to provide an insight into the mechanism by which BPA affects placental development to cause pregnancy complications.

## Impacts of BPA on placental morphology

It has been reported that the placentae of mice exposed to BPA showed a reduction in the decidual basalis, labyrinth, spongiotrophoblast, and/or parietal giant cell layers [28–30] and exhibited impaired spiral artery remodeling [31]. Such morphological alterations are attributable to changes in the expression of genes and the activities of the proteins that control trophoblast proliferation, migration, invasion, fusion, and apoptosis, which are the critical determinants of placental morphology, and are described in more detail below. Therefore, the identification of such molecules is crucial.

One candidate gene that could be associated with BPA-induced defective placental morphology is achaete-scute complex homolog (*Ascl2*), which is a maternally imprinted gene in the imprinting center 2 cluster. Several rodent knockout models have shown that *Ascl2* is required for the formation of the various placental cell layers [32–35]. Exposure of female mice to a relatively high dose of BPA (10 mg/kg body weight per day, which is below the low observed adverse effect level of exposure) [36, 37] but not a lower dose (10 μg/kg body weight per day) for 14 days before conception and up to 12.5 days after conception significantly reduced genome-wide methylation and induced a biallelic expression of *Ascl2* in the placenta. Histological analysis revealed that these epigenetic alterations resulted in abnormal placental development, including larger total area of the placenta, but smaller labyrinth/placenta ratio [38]. However, in a further study in which BPA administration (200 μg/kg body weight) to mice within the same duration disrupted the placentae, no change in *Ascl2* expression was detected via RNA sequencing analysis, although there was a modest decrease in its mRNA levels according to qPCR analysis [30]. Therefore, it remains to be validated whether the BPA-mediated degeneration of the placenta is through aberrant expression of *Ascl2*.

### BPA and trophoblast proliferation

Both hypo- and hyperproliferative rates of trophoblasts impair normal placental development. For instance, when CTB proliferation is reduced, less CTBs become available to undergo differentiation, leading to defective STB formation and EVT invasion. Similarly, when proliferation is exaggerated, trophoblast differentiation becomes inhibited to impair STB formation and EVT invasion.

Trophoblast proliferation is preceded by cell division. The process between successive cell divisions is known as the cell cycle. The cell cycle is subdivided into different phases: G1 phase, S phase, G2 phase, and the M phase. The progression of the cell cycle is enhanced by the timely expression of several molecules such as proliferating cell nuclear antigen (PCNA), MKI67, c-MYC, and the cyclins as well as the activation of several signaling pathways. Different studies that assessed the impact of BPA on trophoblast proliferation yielded discordant results (Table 1) [39–46]. From these results, the effect of BPA on trophoblast proliferation is seemingly concentration- and cell-dependent and that our knowledge of the actual impact of BPA on trophoblast proliferation is incomplete. Therefore, future studies are recommended to investigate how varying concentrations of BPA affect the expression of proliferation-associated signaling pathways and the proportion of cells at each phase of the human trophoblast cell cycle. This will help validate the actual impact of BPA on human trophoblast cell proliferation.

**Table 1:** The impact of BPA on the proliferation of different trophoblast models.

Experimental model	BPA dose	Exposure duration	Impact on proliferation	Impact on proliferation markers	Reference
1. JEG3 cells	10^−8^, 10^−7^, and 10^−6^ M	48 h	Decreased	1) Decreased c-MYC expression 2) Did not affect cyclin D1 expression	39
2. JEG3 cells	0.01–100 μM	24 h	Decreased	None measured	40
3. HTR8/SVneo cells	1 nM	24 h	Decreased	Decreased PCNA expression	41
4. HTR8/SVneo cells	10^−15^, 10^−13^, 10^−11^, 10^−9^, 10^−7^, and 10^−5^ M	24 h, 48 h, and 72 h	No effect	No change in PCNA expression	42 and 43
5. Bewo cells	<1 and 100 μM	48 h	No effect	None measured	44
6. Bewo cells	1 and 10 μM	48 h	Increased	None measured	44
7. Bewo cells	1000 Μm	48 h	Decreased	None measured	44
8. Bewo cells	50 nM	72 h	No effect	None measured	45
9. Mice	4 μM (82.5 μg/kg/day)	14 days	No effect	No change in *Ki67* expression	46

### BPA and trophoblast migration and invasion

Differentiation of CTBs into DCTs, which migrate and attach to the decidua and eventually invade the uterus as EVTs, is a crucial event. This event is facilitated by the downregulation of epithelial molecules such as E-cadherin, the upregulation of mesenchymal molecules such as vimentin and N-cadherin, the increased activities of several enzymes such as the matrix metalloproteinases (MMPs), the inhibition of the activities of several enzymes such as tissue inhibitors of metalloproteinases (TIMPs) and DNA methyltransferases (DNMTs), and the activation of several signaling pathways such as the extracellular signal-regulated protein kinase 1 and 2 (ERK-1/2) pathway.

The effect of BPA on trophoblast invasion has also been investigated with different experimental approaches. In one of these approaches, the effects of BPA on the invasive ability of BeWo cells and its possible mechanism were determined. The BeWo cells were treated with BPA and were co-cultured with human endometrial cells to mimic embryo implantation in a transwell model. The results showed that daily treatment of the cells with different BPA concentrations reduced their invasiveness. Treatment of the cells with 0.01, 1, and 100 μM of BPA for 48 h resulted in a decrease in invasion by 0.04%, 16.48%, or 49.92%, respectively, when compared to controls. However, the impacts of these concentrations on E-cadherin and DNMT-1 were different. BPA at 100 and 1 μM decreased the mRNA levels of E-cadherin, whereas BPA at 0.01 μM increased it. These concentrations were also able to increase the mRNA level of DNMT-1; however, only the 100 μM mediated increase was significant compared to the controls. BPA at 10, 1, and 0.1 μM were able to increase the protein level of E-cadherin, but BPA at 100 μM decreased it. Similarly, BPA at 10, 1, and 0.1 μM were able to decrease the protein level of DNMT1, but BPA at 100 μM increased it. Moreover, each BPA concentration changed the balance of MMPs/TIMPs in the cells by downregulating MMP-2 and MMP-9 and upregulating TIMP-1 and TIMP-2 at the protein level [44]. The fact that—regardless of the discrepancies in the impact of the various BPA concentrations on E-cadherin, DNMT-1, MMP, and TIMP expression—there was an overall inhibition of invasion indicates that BPA is able to reduce the invasiveness of BeWo cells in a concentration-dependent manner.

When HTR-8/SVneo cells were exposed to BPA concentrations at 10^−15^, 10^−13^, 10^−11^, 10^−9^, and 10^−7^ M for 24, 48, and 72 h, their rate of migration and invasion were reduced. This effect was highly exhibited by the 10^−13^ and 10^−11^ M concentrations, and this was directly proportional to the duration of exposure. Co-culture studies with human umbilical cord endothelial cells (HUVEC) showed that trophoblast-endothelial interactions, which are subsets of trophoblast invasion, were not affected by each BPA concentration. However, each concentration was able to enlarge the nuclei of the cells via the upregulation of p57^Kip2^ [42], which is a molecule that is able to arrest the cell cycle to promote trophoblast differentiation [47]. This indicates that BPA is able to induce differentiation of the cells toward polyploidy by the process of endoreduplication. In another study involving HTR-8/SVneo cells, it was found that BPA (10^−8^–10^−6^ mol/l) significantly reduced the migration and invasion of the cells after 48 h of treatment. BPA at 10^−6^ mol/l decreased MMP-2 and MMP-9 levels, but increased TIMP-1 and TIMP-2 levels at concentrations of 10^−7^–10^−6^ mol/l, indicating that BPA is able to disrupt the MMP/TIMP balance. Also, BPA (10^−7^ mol/l) decreased integrin α5/β1 and vimentin levels but increased CD-97 levels. However, E-cadherin, N-cadherin, and occludin levels remained unchanged upon BPA treatment. All these inhibitory effects were elicited via the G protein-coupled receptor 30 (GPR-30) [48].

In a similar study, BPA at 10 μM decreased both JEG3 spheroids outgrowth and invasion after 24 h of treatment. The mRNAs of MMP-2 and MMP-9 were reduced, while the mRNAs of TIMP-1 and plasminogen activator inhibitor type 1 (PAI-1) were increased. Also, the TIMP-1 level in the serum-free culture medium was increased. The BPA-decreased spheroid outgrowth and invasion could be reversed by IC I182780 (an antagonist of ERs), but not with G15 (an antagonist of GPR-30) [40]. These findings show that the inhibitory effect of BPA on JEG3 spheroid outgrowth and invasion was elicited through canonical ERs.

Trophoblast invasion into the uterus also depends on the expression of certain molecules in the decidua, which can contribute to promoting or inhibiting trophoblast invasion. It was found that 1–10 μM of BPA was able to reduce the ability of decidual cells to attract invading HTR-8/SVneo cells and human primary trophoblast cells, after 48 h of treatment. This occurred as a result of the ability of BPA to inhibit the expression of C-X-C motif chemokine ligand 8 (CXCL-8) in the decidual stromal cells (DSCs) via an increase in AKT, p38, and ERK phosphorylation in the DSCs. It was also observed that the BPA-augmented phosphorylation of ERK-1/2 and inhibition of CXCL8 expression occurred through GPR-30 and ESR1/2 [49]. Also, treatment of decidualized cells with 10 μM of BPA for 24 h showed decreased JEG3 spheroid outgrowth and invasion, along with the downregulation of MMP-2 and MMP-9 and the upregulation of PAI-1 and tumor necrosis factor alpha (TNF-α) [40]. However, in a similar study, when decidual cells were treated with 10 μM of BPA for 24 h, there was an increase in the expression of LIF and a decrease in the expressions of anti-invasion molecules including interleukin-10, PAI-1, and TNF-α to promote JEG-3 spheroid outgrowth and invasion [50]. The reason behind these conflicting reports is difficult to deduce because similar experimental conditions were seemingly applied in both studies. It should be kept in mind that for BPA studies, even variation in plastic ware used for cell culture can affect the results, as BPA contamination from cell culture dishes was found to be variable across different manufacturers [51].

In an in vivo study, 4uM (82.5 μg/kg/day) of BPA administration for 14 days resulted in the development of preeclampsia-like features in mice. These features were associated with decreased trophoblast cell invasion, increased retention of smooth muscle cells and a decrease in the vessel areas at the junctional zone of the placenta. This occurred as a result of the upregulation of *TIMP-1, TIMP-2,* and DNMT-1, and the downregulation of *MMP*-2, *MMP*-9*, β-catenin*, and the Wnt family member 2 (*WNT-2*)*.* Furthermore, BPA impeded the interaction of HTR-8/SVneo cells with HUVEC cells, and downregulated *WNT-2* via increased DNA methylation [46].

**Figure 1:**
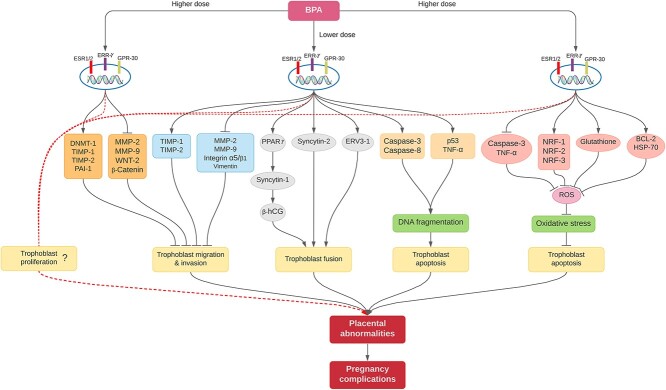
Proposed mechanisms of how BPA affects placentation. Both higher and lower doses of BPA are able to elicit different effects on trophoblast cells during placentation. These doses induce their effects through the nuclear receptors: ESR1/2, ERRγ-1, and/or GPR-30. A higher BPA dose upregulates DNMT-1, TIMP-1, TIMP-2, and PAI-1, while downregulating MMP-2, MMP-9, WNT-2, and β-catenin to inhibit trophoblast invasion. Also, a lower BPA dose upregulates TIMP-1 and TIMP-2, and downregulates MMP-2, MMP-9, integrin α5/β1, and vimentin to inhibit trophoblast invasion. The lower dose again increases sycytin-1 expression via the upregulation of PPARγ to promote β-hCG secretion. Furthermore, this dose augments syncytin-2 and ERVW3-1 expression. These actions result in the exaggeration of trophoblast fusion at the expense of trophoblast invasion. Moreover, lower doses of BPA induce trophoblast apoptosis by increasing caspase-3 and caspase-8 activities and upregulating p53 and TNF-α to facilitate DNA fragmentation, thus increasing trophoblast apoptosis. However, in trophoblast cells which are under oxidative stress, higher doses of BPA decrease caspase-3 activity, increase TNF-α, NRF-1, NRF-2, and NRF-3 expression, increase the levels of glutathione, and augment the expression of BCL-2 and HSP-70 to reduce the levels of ROS. Consequently, oxidative stress is decreased in these cells, and apoptosis is inhibited. The actual impact of BPA on trophoblast proliferation is yet to be validated, due to the conflicting nature of the available reports. Taken together, all these interferences of the different doses of BPA in trophoblast cell fate decisions induce abnormal placentation to trigger pregnancy complications. We considered <1 μM and ≥1 μM of BPA as lower doses and higher doses, respectively. Abbreviations: BPA, bisphenol A; ESR1/2, estrogen receptor; ERRγ-1, estrogen-related receptor γ 1; GPR-30, G protein-coupled receptor 30; MMP-2, metalloproteinase 2; MMP-9, metalloproteinase 9; WNT-2, Wnt family member 2; DNMT-1, DNA methyltransferase 1; TIMP-1, tissue inhibitors of metalloproteinase 1; TIMP-2, tissue inhibitors of metalloproteinase 2; PAI-1, plasminogen activator inhibitor type 1; PPARγ, peroxisome proliferator-activated receptor γ; β-hCG, beta human chorionic gonadotropin; ERV3-1, endogenous retrovirus group 3 member *1*, envelope; TNF-α, tumor necrosis factor alpha; NRF-1, nuclear respiratory factor 1; NRF-2, nuclear respiratory factor 2; NRF-3, nuclear respiratory factor 3; BCL-2, B-cell lymphoma-2; HSP-70, heat shock proteins; ROS, reactive oxygen species; Symbols*:* (→), increase or activate; (⊥), decrease or inhibit; (- - - - -, ?), undefined.

Although these studies involved different BPA concentrations and different trophoblast models, they generally indicate that BPA has the tendency of promoting DNA methylation, reducing the MMP/TIMP ratio, and increasing the expression of other anti-invasion molecules in trophoblast cells to inhibit their migration and invasion (Figure 1).

### BPA and trophoblast fusion

Trophoblast fusion, also called trophoblast syncytialization, is the aggregation of the CTB cells, the disappearance of their adjacent membranes, and the mixing of their protoplasm to form a syncytium called the STB. Downregulation of molecules such as E-cadherin and the upregulation of molecules such as peroxisome proliferator-activated receptor γ (PPARγ), glial cell missing 1 (GCM1), beta-human chorionic gonadotropin (β-hCG), and the endogenous retroviruses (ERVs) enhance trophoblast fusion.

To the best of our knowledge, only a single study has reported the impact of BPA on trophoblast fusion. In that study, Narciso and colleagues exposed BeWo cells to 50 and 1 nM of BPA for 48 and 72 h, and then assessed the mRNA and protein expression of ERVW-1 (syncytin-1), ERVFRD-1 (syncytin-2), and ERV3-1. They also measured the effects of BPA on the secretion of β-hCG and the expression of GCM-1. At 50 nM, BPA induced the expression of syncytin-1, syncytin-2, ERV3-1, and PPARγ. There was also an increase in the secretion of β-hCG. All these resulted in the promotion of BeWo cells fusion, as evidenced by an increase in the fusion index of the cells at both durations. The effects were stronger at 72 h than at 48 h. No effect was observed when the cells were treated with 1 nM of BPA. Also, none of the BPA concentrations affected GCM-1 expression at both durations [45]. It has been found that PPARγ induces trophoblast fusion and β-hCG secretion [52–54] via the upregulation of syncytin-1. This PPARγ-mediated increase in syncytin-1 expression can occur directly by interacting with a PPAR responsive element on the ERVW-1 gene [52] and, indirectly, through GCM-1 [52, 54, 55]. This finding means that BPA may augment trophoblast fusion by upregulating syncytin-1 and increasing β-hCG secretion through PPARγ (Figure 1). With placentation bound to occur physiologically, the presence of BPA in the trophoblasts might exaggerate trophoblast fusion to the detriment of trophoblast invasion. This hypothesis though remains to be tested.

### BPA and trophoblast apoptosis

Apoptosis is a common occurrence during placentation, and it is exacerbated in the placentae of complicated pregnancies. In spite of this, the apoptotic mechanisms that occur in the placenta have not been fully clarified. It has been observed that trophoblasts, just as other mammalian cells, express a number of apoptotic signaling molecules such as TNF-α [56] and caspases as well as anti-apoptotic molecules such as heat shock proteins (HSPs) [57] and the B-cell lymphoma-2 (BCL-2) family members [56]. The impact of BPA on trophoblast apoptosis has been investigated to some extent.

Exposure of primary CTBs to BPA concentrations similar to those found in the circulation of pregnant women (from 0.02 to 0.1 μg/ml) stimulated significant apoptosis after 24 h of treatment—as evident in an increase in the number of cells positive for the M30 antibody. There was an increase in the release and the activation of cytosolic adenylate kinase (AK), which was 1.3–1.7 times more than in the nontreated controls. This effect was dose-dependent, starting from as low as 0.0002 to 0.02 μg/ml of BPA [58]. Such increased release of AK is a mark of cell membrane damage. Also, BPA was found to control TNF-α gene expression. At levels ranging between 0.0002 and 0.02 μg/ml, BPA remarkably increased TNF-α expression and secretion. However, higher concentrations of BPA did not affect TNF-α mRNA expression, but were associated with a significant decrease in the production of TNF-α protein [58]. These observations suggest that moderate levels of BPA can induce CTB apoptosis via TNF-α signaling, while higher levels may decrease it.

When JEG3 cells were treated with a range of concentrations of BPA for 48 h, their viability was unaffected by the different BPA concentrations tested (10^−10^–10^−6^ M) indicating that, under these concentrations, BPA did not lead to cell death. However, at 10^−5^ M, BPA induced cytotoxic effects and cell death [39]. These results somehow conflict with the findings of a study that involved the use of human third trimester trophoblast cells. In that study, a BPA concentration of 10^−9^ M was able to induce cell death after 24 h of treatment [58]. These inconsistent findings could be due to the fact that both cell types do not equally express the specific BPA receptor: estrogen-related receptor γ 1 (ERRγ-1). The low levels of ERRγ-1 in JEG3 cells, as compared to the levels in primary CTB and EVT cells [39], suggest that the integrity of ERRγ-1 expression could be a strong determinant of the susceptibility of the third trimester placenta to the toxic effects of BPA.

In a further investigation, a 48 h treatment of JEG3 cells with 10^−8^ and 10^−7^ M of BPA resulted in a significant induction of DNA fragmentation in the cells. Even after 8 h, both concentrations of BPA significantly induced the expression of p53 mRNA, with the effects being directly proportional to the various concentrations [39]. In another study, when BeWo cells were exposed to 50 nM of BPA, there was no effect on the activities of caspase-3, caspase-9, and total poly (ADP-ribose) polymerase (PARP), although a slight increase in the activity of caspase-8 was observed at 12 h posttreatment. However, 1 nM of BPA significantly induced caspase-3 activity at 72 h posttreatment [45]. This observation is consistent with the observation made in a previous study in which an exposure of first trimester chorionic villous explants to 1 nM of BPA for 48 h augmented the apoptotic rate of these cells via an increase in caspase-3 expression [59] and possibly an increase in caspase-3 activity.

Interestingly, in another study that involved the use of BeWo cells and JEG3 cells which were under oxidative stress, BPA treatment for 72 h was rather found to be protective against apoptosis. 1–9 μM of BPA were able to increase glutathione production, reduce intracellular reactive oxygen species (ROS) levels, and increase ATP production [60]. Each of these treatments also led to an increase in the expression of BCL-2 and HSP-70 and a reduction in caspase-3 activity in a concentration-dependent manner. Moreover, these treatments could increase the expression of nuclear respiratory factor (NRF) 1, 2, and 3 [60], which are transcription factors that are known to control trophoblast antioxidant status [61]. These resulted in the activation of the antioxidant response element and subsequent downstream antioxidant enzymes, thus inhibiting cell death [60]. This finding suggests that during trophoblast oxidative stress, BPA could be protective against apoptosis.

These reports indicate that BPA can trigger apoptotic or anti-apoptotic signals in different cells at varying concentrations and exposure durations. The lower concentrations might stimulate elimination of trophoblast cells, whereas higher concentrations can inhibit the physiological removal of superfluous, infected, damaged, and/or dysfunctional trophoblasts. Each of these occurrences can impair placentation (Figure 1).

**Table 2:** The effects of BPA on the mRNA expression of nuclear and some nonreceptors in murine placentae with male or female embryos.

Receptor	Placentae with female embryos	Placentae with male embryos
*Nuclear receptor*		
Estrogen receptor 1 (*ESR1*)	No effect	Decreased
Progesterone receptor (*Pr*)	Decreased	Increased
Liver X receptor (*Lxrα*)	No effect	Increased
Anti-chicken ovalbumin upstream promoter transcription factor (*Coup-Tfα*)	Increased	Decreased
Germ cell nuclear factor	Increased	Decreased
*Nonreceptor*		
Probasin	Increased	Decreased
RNA-specific adenosine	Increased	Decreased
Adam25/testase 2	Increased	Decreased
α-Fetoprotein	Decreased	Decreased
Kinesin light chain 1	Decreased	Decreased
Fast skeletal troponin C	Increased	Decreased

## Impacts of BPA on placental metabolic state

BPA is able to alter the metabolic state of the placenta. Administration of BPA (200 μg/kg body weight) to female mice for 14 days prior to conception and up to 12.5 days after conception reduced the concentrations of estradiol, d-fructose, and the metabolites docosahexaenoic acid (DHA), sophorose (2-O-β-d-glucopyranosyl-d-glucose), and glycolic acid in the placentae [30]. The same BPA dose reduced the concentration of 5-hydroxytryptamine receptors (5-HT), increased that of its immediate metabolite *5*-hydroxyindoleacetic acid, and raised the concentration of dopamine but had no effect on the levels of gamma-aminobutyric acid (GABA) as well as on the transcript levels of dihydroxyphenylalanine, tyrosine hydroxylase, tryptophan hydroxylases, and dopa decarboxylase which are involved in the biosynthesis of 5-HT and dopamine in the placentae. The transcript levels of genes such as catechol-*O*-methyl transferase and monoamine oxidases, which metabolize 5-HT and dopamine, were also not affected in these placentae [30].

There is evidence that BPA is soluble in lipids, can shuttle between the mother and fetus via passive diffusion, mainly in an unconjugated form [59, 62], and can affect the intraplacental transport of glucose and other molecules by regulating the expression of some of their transporters. Glucose has numerous transporters. Among them, glucose transporter type 1 (GLUT-1) and glucose transporter type 4 (GLUT-4) are the most important transporters of glucose through the placenta. Both transporters are expressed in the STB [63, 64] and have crucial roles in sustaining glucose homeostasis during placentation and fetal development [64]. GLUT-1 expression was found to be increased after 48 h in BPA-treated (1 nM and 1 μM) villous explants from normal weight women but decreased in those from overweight women. No effect was elicited by BPA on GLUT-4 expression [65]. In a further study, treatment of HTR-8/SVneo cells with BPA (1 nM) for 48 h led to an increase in GLUT-1 expression and elevated glucose uptake in the BPA-treated cells than in the controls [66]. These observations suggest that BPA can increase the quantity of glucose that traverses the placenta to the fetus to induce fetal hyperglycemia and cause associated developmental defects.

Some nuclear receptors serve as metabolic receptors, and help animals to adapt to environmental changes by modulating the expression of metabolic genes and pathways [67]. Sexually dimorphic differences have been reported as to how BPA affects select nuclear receptors (Table 2) [68]. The change in estrogen receptor 1 (ESR1) expression may affect the sensitivity of the placentae to endogenous 17β-estradiol, and hence indirectly elicit an estrogenic effect in these placentae. The alteration in liver X receptor (*Lxrα*) expression may disrupt steroid metabolism in the placenta of male embryos to affect its formation and functioning, since it is known to regulate physiological processes such as lipid metabolism, adipogenesis, and immunity [69, 70]. Also, the change in anti-chicken ovalbumin upstream promoter transcription factor 1 (*Coup-Tfα*) expression (see Table 2) might affect hormonal homeostasis and differentiation of placental cells, since this gene is known to play similar roles in other parts of the body [71, 72].

The impact of BPA on the expression of some nonreceptors also differs between placentae with female embryos and placentae with male embryos (Table 2) [68]. The differential impact of BPA on probasin suggests that BPA exhibits sexually dimorphic androgenic effects on mouse placentae. Since RNA-specific adenosine deaminase catalyzes the conversion of adenine into inosine [73], differential expression of this gene implicates BPA in the editing of mRNAs within the placenta. It is known that α-fetoprotein binds to estrogens in mice and controls estrogenic activities [74]. Therefore, the downregulation of α-fetoprotein can influence the quantity of estrogen that reaches the embryo to control its development. The impact of BPA on kinesin light chain-1 and fast skeletal troponin C indicates that BPA may affect placental cytoskeletal organization, which is a critical regulator of trophoblast migration and intracellular transport during placentation. Nonetheless, the actual roles of these endogenous biochemical molecules in placentation are yet to be determined. The embryo-sex-dependent differences in gene expression point to some likely novel mechanisms of BPA toxicity, including potential feminization of male embryos. If so, such changes would be similar to those observed in fish where BPA and other EDC exposure increased vitellogenin (VTG, a protein normally expressed by females) in males [75]. As such, VTG is considered a biomarker of EDC exposure in many fish species. It is possible that some of the genes listed above may serve as a barometer of exposure in mammals.

Metabolic state is also dependent on cytokines, as pro- and anti-inflammatory cytokines are able to activate opposing metabolism-modulating activities during microbial infection [76]. It has been found that soon after embryo implantation, there is a strict regulation of cytokine production to enhance placentation and embryonic development, and that disturbances in this regulatory network by infection or other sources of inflammation can increase the risk for pregnancy complications [7, 8, 10, 12]. Treatment with BPA (≤10 000 nM) for 24 h increased the basal production of proinflammatory cytokines (interleukin-1β and interleukin-6) and the oxidative stress biomarker (8-isoprostane) in human trophoblast explants but had minimal effects on bacteria-stimulated production of these cytokines and marker in the explants in a dose-dependent manner [77]. These data suggest that during placentation, BPA can exaggerate the physiological secretion of cytokines, but in the event of bacterial infection in the placenta, this chemical may have no effect on the infection-mediated secretion of the cytokines.

## Conclusions

The above reports indicate that BPA exposure leads to pathological changes in the murine placenta, and these likely have transcriptomic, hormonal, and metabolic underpinnings. We speculate that during pregnancy, the accumulation of BPA in the trophoblasts exaggerates STB formation while inhibiting EVT formation such that shallow trophoblast invasion would occur. These concentrations also promote trophoblast apoptosis. However, at higher concentrations, BPA inhibits apoptosis—especially in trophoblast cells which are under oxidative stress. There is no clear consensus though on how BPA affects trophoblast proliferation. As a xenobiotic, BPA is not subject to normal homeostatic mechanisms, although, in adults, it can be metabolized by the liver to less potent forms. Therefore, its presence at the feto-maternal interphase has the potential to induce abnormal placentation, regardless of the concentration, leading to pregnancy complications in humans (Figure 1).

## Future perspectives

Since BPA and its products have not been banned worldwide, its global consumption will continue to rise dramatically in the coming decades. Accordingly, epidemiological studies must continue to monitor the concentration of BPA in the blood and placentae of pregnant women. Experimental rodent models and in vitro studies may need to adjust exposure dosages to reflect those currently observed in the general populace. Utilizing additional model systems, such as human trophoblast stem cells [78] or trophoblast organoids [79, 80], is likely to enhance our understanding of how BPA affects the placenta and potentially helps reconcile conflicting reports. Additionally, confirmation of the proposed mechanisms, the identification of the specific nuclear receptors that mediate the placental actions of BPA, and investigation of the mechanisms by which each aforementioned transcript/protein, hormone, or metabolite mediates the BPA-induced impairment of placentation are needed. This will enhance a detailed and a clear understanding of the mechanism of BPA toxicity in the placental microenvironment. Such findings might also be used as the basis for early diagnostic and remediation approaches.

## Author contributions

EAA-G wrote the initial draft. GT and CSR contributed to critical review of the draft and revising the manuscript. All authors approved the final manuscript.

## Data availability

All data referred to in this article can be obtained through the original cited publications.
